# Supplementary material for: Long-term ex situ normothermic perfusion of human split livers for more than 1 week
Source: Nat Commun. 2023 Aug 8;14:4755. doi: 10.1038/s41467-023-40154-8 (PMC10409852; doi:10.1038/s41467-023-40154-8)
Supplement: Supplementary file 1 — Supplementary Information [file 41467_2023_40154_MOESM1_ESM.pdf]

TITLE:

Long-term ex-situ normothermic perfusion of human split livers for more than 1 week

Supplementary Information

Supplementary Table 1: Liver characteristics, donor liver function tests and donation details

CIT: cold ischaemic time, WCRS: withdrawal of cardiorespiratory support, WIT: warm ischaemic time

**Liver number**	**Bilirubin (µmol/L)**	**AST (U/L)**	**ALT (U/L)**	**ALP (U/L)**	**GGT (U/L)**	**INR**	**Time to death (WCRS to cessation of circulation) (minutes)**	**CIT (minutes)**	**WIT (systolic blood pressure <50 to cold flush) (minutes)**
1	3	63	63	42	32	1.1	N/A	238	N/A
2	4	56	43	70	150	1.1	19	305	20
3	9	19	21	71	65	1.4	31	449	29
4	6	40	50	70	68	1	21	456	22
5	7	110	30	88	38	1.4	N/A	187	N/A
6	152	95	241	246	333	2.1	N/A	430	N/A
7	9	146	187	82	51	1.1	36	284	19
8	6	53	25	228	308	1	19	272	29
9	11	51	46	68	108	1.5	17	274	14
10	29	49	51	117	79	2	N/A	397	N/A

Supplementary Table 2: Perfusion details after long-term normothermic perfusion of partial human livers

ERG: extended right graft, LLSG: left lateral segment graft

Liver number	Whole Liver weight (g)	Partial liver	Weight after split (g)	Weight at end (g)	Maximum hepatocellular viability* (hours)	Liver survival (hours)
1	1862	LLSG	437	438	22	159
		ERG	1501	1445	21	39
2	2188	LLSG	626	595	171	209
		ERG	1514	1440	210	228
3	2407	LLSG	575	477	87.5	107
		ERG	1771	1470	262	288
4	1279	LLSG	238	252	88.5	108
		ERG	846	895	139.5	167
5	1615	LLSG	227	353	125	181
		ERG	1210	1302	125	163
6	1500	LLSG	279	362	82	96
		ERG	1113	1413	66	79
7	1343	LLSG	241	313	90	114
		ERG	845	902	115	123
8	3633	LLSG	726	738	105.5	150.5
		ERG	2455	2439	160.5	177.5
9	2497	LLSG	418	623	165.5	327.5
		ERG	1522	1719	295.5	327.5
10	1461	LLSG	292	457	201.5	257.5
		ERG	959	1172	190.5	222.5

*Viability according to the criteria proposed by the VITTAL clinical trial (≤2.5mmol/L, and two or more of: bile production, pH≥7.30, glucose metabolism, hepatic arterial flow ≥150ml/minute and portal vein flow ≥500ml/minute, or homogeneous perfusion).[1]

Supplementary Table 3: Assessment of hepatocellular and hepatobiliary viability of partial livers during normothermic machine perfusion

ERG: extended right graft, LLSG: left lateral segment graft

Liver number	Partial liver	Hepatocellular viability* duration and criteria causing failure	Overall survival duration and criteria causing failure	Hepatobiliary viability# duration and criteria causing failure, bile pH
1	LLSG	22 hours<br>Lactate 3.43	159<br>Lactate 12.83	21.5 hours<br>Lactate 1.93<br>Bile pH Nil
	ERG	21 hours<br>Lactate 15.08	39 hours<br>Lactate 13.3	20.5 hours<br>Lactate 1.72<br>Bile pH Nil
2	LLSG	171 hours<br>Lactate 2.84	209 hours<br>Lactate 12.59	100 hours<br>Lactate 2.54<br>Bile pH 7.795
	ERG	210 hours<br>Lactate 5.18	228 hours<br>Lactate 19.08	207 hours<br>Lactate 2.11<br>Bile pH 7.546
3	LLSG	87.5 hours<br>Lactate 2.97	107 hours<br>Lactate 11.67	87.5 hours<br>Lactate 2.97<br>Bile pH 7.649
	ERG	262 hours<br>Lactate 3.08	288 hours<br>Lactate 11.2	163 hours<br>Lactate 1.82<br>Bile pH 7.478
4	LLSG	88.5 hours<br>Lactate 2.50	108 hours<br>Lactate 11.98	79.5 hours<br>Lactate 1.79<br>Bile pH 7.6
	ERG	139.5 hours<br>Lactate 2.84	167 hours<br>Lactate 3.78<br>pH 6.90, glucose <2	132.5 hours<br>Lactate 2.08<br>Bile pH 7.412
5	LLSG	125 hours<br>Lactate 3.75	181 hours<br>Lactate 14.89	110 hours<br>Lactate 1.76<br>Bile pH 7.662
	ERG	125 hours<br>Lactate 3.95	163 hours<br>Lactate 14.98	123 hours<br>Lactate 2.31<br>Bile pH 7.658
6	LLSG	82 hours<br>Lactate 4.47	96 hours<br>Lactate 0.57<br>pH 7.127, glucose 1.9	82 hours<br>Lactate 4.47<br>Bile pH 7.493
	ERG	66 hours<br>Lactate 2.96	79 hours<br>Lactate 6.69<br>Arterial flow 80ml/min, non-homogeneous perfusion	60 hours<br>Lactate 2.3<br>Bile pH 7.786
7@	LLSG	90 hours<br>Lactate 2.89	114 hours<br>Lactate 11.87	82 hours<br>Lactate 2.22<br>Bile pH N/A (clamped)
	ERG	115 hours<br>Lactate 3.1	123 hours<br>Lactate 19.64	107 hours<br>Lactate 2.0<br>Bile pH N/A (clamped)

8	LLSG	105.5 hours<br>Lactate 2.54	150.5 hours<br>Lactate 19.8	94.5 hours<br>Lactate 2.77<br>Bile pH 7.48
	ERG	160.5 hours<br>Lactate 2.98	177.5 hours<br>Lactate 13.07	156.5 hours<br>Lactate 2.21<br>Bile pH 7.419
9	LLSG	165.5 hours<br>Lactate 2.93	327.5 hours<br>Lactate 13.31	101.5 hours<br>Lactate 1.81<br>Bile pH 7.445
	ERG	295.5 hours<br>Lactate 3.61	327.5 hours<br>Lactate 16.98	295.5 hours<br>Lactate 3.61<br>Bile pH 7.778
10	LLSG	201.5 hours<br>Lactate 3.56	257.5 hours<br>Lactate 0.84<br>pH low, arterial flow 109ml/min	112.5 hours<br>Lactate 1.9<br>Bile pH 7.761
	ERG	190.5 hours<br>Lactate 2.61	222.5 hours<br>Lactate 10.07	188.5 hours<br>Lactate 1.72<br>Bile pH 7.541

*Hepatocellular viability according to the criteria proposed by the VITTAL clinical trial (≤2.5mmol/L, and two or more of: bile production, pH≥7.30, glucose metabolism, hepatic arterial flow ≥150ml/minute and portal vein flow ≥500ml/minute, or homogeneous perfusion).[1]

#Hepatobiliary viability according to the criteria used in the DHOPE-COR-NMP clinical trial (lactate <1.7mmol/L, pH 7.35-7.45, bile production >10ml and bile pH >7.45).[2]

@ No bile pH measured for Liver 7 due to haemobilia requiring clamping of the bile duct.

Supplementary Table 4: Donor characteristics and liver survival

DCD: donation after circulatory determination of death

Donor characteristic	Liver survival ≤7 days (n=11)	Liver survival >7 days (n=9)	p-value
Male sex	7/11	7/9	p=0.642
Age (mean ± standard deviation)	53.55 ±15.38	52.78 ±13.33	p=0.908
DCD	6/11	6/9	p=0.670
Donor BMI (median, 25%-75% percentiles)	29.0 (23.5-43.6)	25.2 (22.8-30.7)	p=0.293

Supplementary Figure 1: Long-term normothermic machine perfusion of human whole livers. (A) Perfusate lactate levels during perfusion demonstrating rapid clearance followed by maintenance of low lactate levels until the point of organ failure. (B) Overall survival times demonstrating that all livers (3/3) survived >7 days.

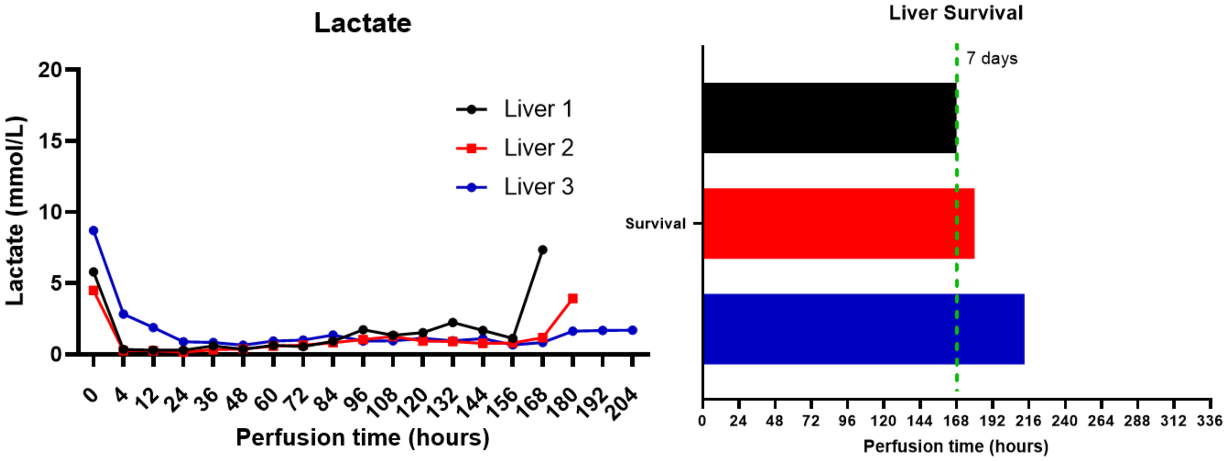

Supplementary Figure 2: Additional biochemical and functional evidence of long-term graft function of human split livers.
Typically, bilirubin (A), and GGT (B) levels in the perfusate remained low during perfusion but gradually increased during perfusion towards the point of graft failure. Urea (C) levels remained consistent throughout perfusion. Prothrombin time (D) shortened as liver synthetic function improved after reperfusion and then lengthened as liver function deteriorated towards the end of perfusion. Albumin (E), total protein (F) and ALP (G) levels remained steady during perfusion. Perfusate levels of ALT peaked in the first 48-72 hours after whole liver reperfusion before plateauing then decreasing during long-term perfusion (H).

ALP: alkaline phosphatase, ALT: alanine amino-transferase, GGT: Gamma-glutamyl transferase

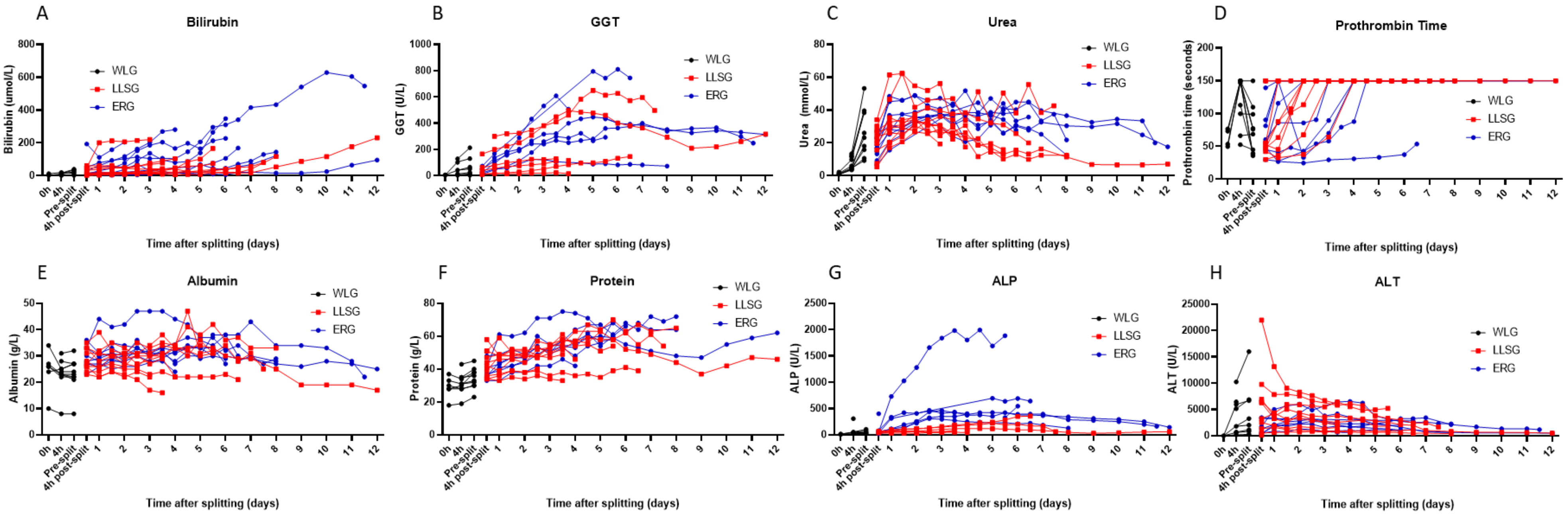

Supplementary Figure 3: Histopathology analysis of bile duct biopsies taken during long-term perfusion of human split livers.
Slides were stained with haematoxylin and eosin to assess biliary architecture and integrity. Mural stromal necrosis was present (A, 9 days after splitting), however intact biliary epithelium was seen in 80% of livers (B, 3 days after splitting) suggesting preserved biliary tree integrity. Star marks mural stromal necrosis and arrows mark intact epithelium.

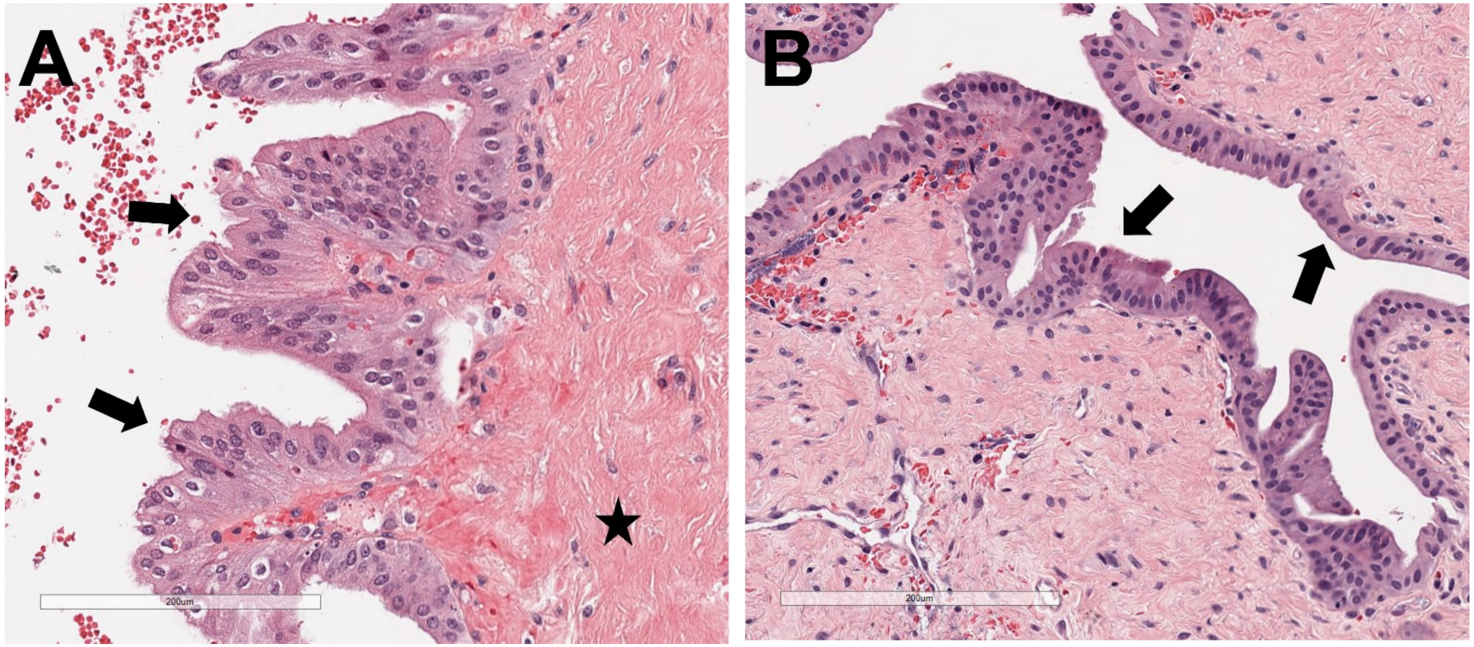

Supplementary Figure 4. Additional factors related to long-term survival of human split livers.
Perfusate bilirubin (A), GGT (B), protein (C), albumin (D), ALP (E), and ALT (F) did not demonstrate significant differences between livers that survived >7 days or ≤7 days. After adjustment for liver weight, hepatic artery flow and portal vein flow demonstrated a less pronounced difference between livers that survived >7 days or ≤7 days (G-H). Hepatic artery flow was significantly higher at 4h in livers that survived >7 days (median 256ml/min [IQR: 228-367ml/min] vs 227ml/min [IQR: 166-238ml/min], p=0.019, Mann-Whitney U Test). All grouped data are presented as median (IQR) except for GGT and ALT which were normally distributed and presented as mean (standard deviation), n=20 partial livers, 9 survived >7 days, 11 survived ≤7 days, *p<0.05.

ALP: alkaline phosphatase, ALT: alanine amino-transferase, GGT: Gamma-glutamyl transferase, IQR: interquartile range

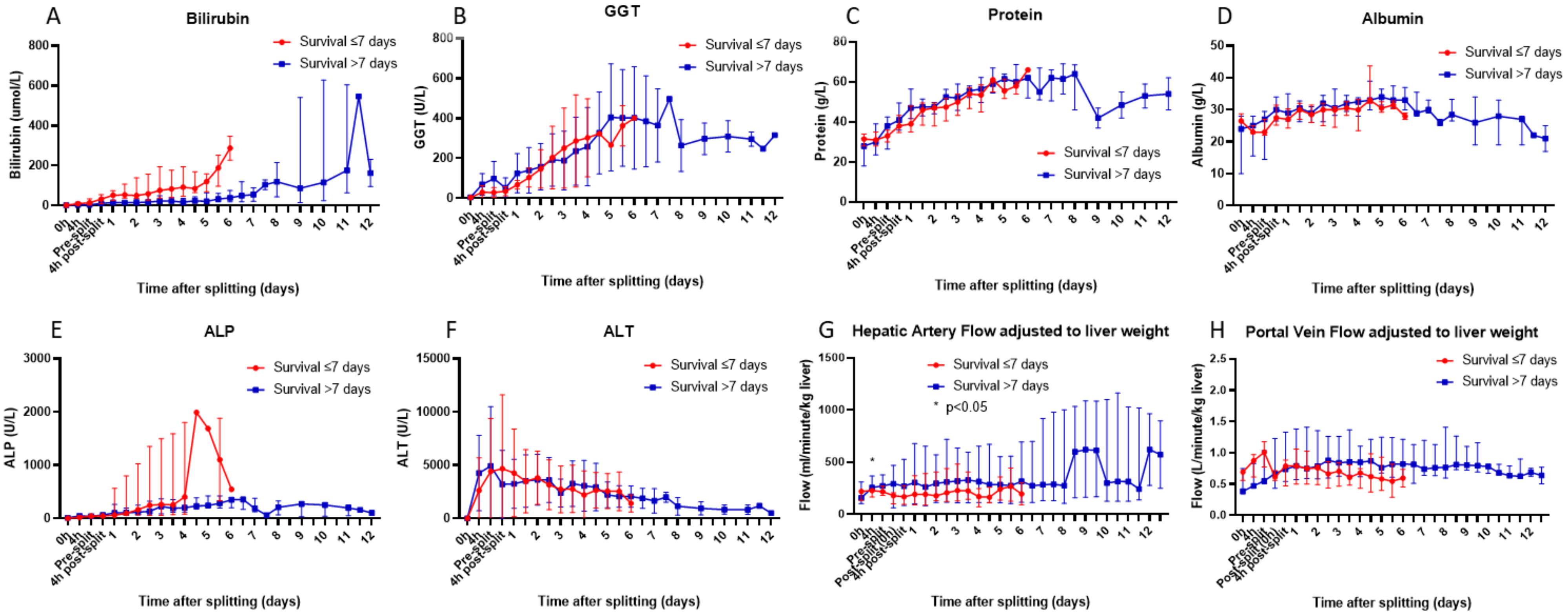

Supplementary Figure 5. Algorithm to guide modification of parameters and maintenance of normothermic machine perfusion

PO2	<150mmHg	Increase arterial FiO2 (air O2 mixer) by 2-3%
	150-180mmHg	No action
	>190mmHg	Decrease FiO2 (air O2 mixer) by 2-3%
Potassium	>5.5mmol/L	Increase dialysis inflow by 50ml/h AND dialysis outflow by 50ml/h
	4.5-5.5mmol/L	No action
	3-4.5mmol/L	Decrease dialysis inflow by 50ml/h AND dialysis outflow by 50ml/h
Haemoglobin	<50g/L	Increase dialysis ouftflow by 20ml/h
	50-60g/L	No action
	>60g/L	Check reservoir volume. If low, decrease dialysis outflow by 20ml/h
pH	>7.3	If pCO2 high, increase arterial ventilation by 0.05ml/min<br>If HCO3 low, increase dialysis by 20ml/h
	7.3-7.45	No action
	>7.45	If pCO2 low, decrease arterial ventilation by 0.05ml/min<br>If HCO3 high, decrease dialysis by 20ml/h
Glucose	<10mmol/L	Increase glucose infusion by 3ml/h<br>Increase glucagon by 1ml/h<br>Decrease insulin by 1ml/h
	10-15mmol/L	No action
	>15mmol/L	Decrease glucose infusion by 3ml/h<br>Decrease glucagon by 1ml/h<br>Increase insulin by 1ml/h

Supplementary Notes 1: Methods for determination of hepatic tissue adenosine triphosphate and glycogen.

Hepatic tissue adenosine triphosphate (ATP) content was determined using the ATP Bioluminescent Kit (FLAA Sigma-Aldrich) according to the manufacturer's instructions. Frozen core biopsies were homogenised and sonificated on ice in 100uL of SONOP Buffer (0.372g EDTA in 130ml dH_2O, adjusted to pH 10.9 with NaOH with ethanol) and centrifuged at 13000g to remove particulates. ATP concentration was calculated using luminosity according to the standards in the manufacturer's instructions. This was corrected for protein concentration which was determined using the BCA Protein Assay Kit (ThermoFisher Scientific) and results expressed as µmol/g protein.

Hepatic tissue glycogen was determined using the Glycogen Assay Kit (MAK016, Sigma-Aldrich) according to the manufacturer's instructions. Frozen core biopsies were homogenised and sonificated on ice in 100µl H_2O before being centrifuged at 13000g to remove particulates. The glycogen concentration was measured according to the manufacturer's instructions and the results were expressed as µg/µg of tissue.

Supplementary References:

1 Mergental, H. *et al.* Transplantation of discarded livers following viability testing with normothermic machine perfusion. Nature communications **11**, 2939, (2020).

2 van Leeuwen, O. B. *et al.* Transplantation of High-risk Donor Livers After Ex Situ Resuscitation and Assessment Using Combined Hypo- and Normothermic Machine Perfusion: A Prospective Clinical Trial. Ann Surg **270**, 906-914, (2019).
