# Supplementary material for: Long-term ex situ normothermic perfusion of human split livers for more than 1 week
Source: Nat Commun. 2023 Aug 8;14:4755. doi: 10.1038/s41467-023-40154-8 (PMC10409852; doi:10.1038/s41467-023-40154-8)
Supplement: Supplementary file 3 — Reporting Summary [file 41467_2023_40154_MOESM3_ESM.pdf]

nature portfolio

Corresponding author(s): Carlo Pulitano, Ngee-Soon Lau

Last updated by author(s): Jul 6, 2023

Reporting Summary

Nature Portfolio wishes to improve the reproducibility of the work that we publish. This form provides structure for consistency and transparency in reporting. For further information on Nature Portfolio policies, see our Editorial Policies and the Editorial Policy Checklist.

Statistics

For all statistical analyses, confirm that the following items are present in the figure legend, table legend, main text, or Methods section.

n/a	Confirmed	
☐	☒	The exact sample size (n) for each experimental group/condition, given as a discrete number and unit of measurement
☐	☒	A statement on whether measurements were taken from distinct samples or whether the same sample was measured repeatedly
☐	☒	The statistical test(s) used AND whether they are one- or two-sided<br>*Only common tests should be described solely by name; describe more complex techniques in the Methods section.*
☐	☒	A description of all covariates tested
☐	☒	A description of any assumptions or corrections, such as tests of normality and adjustment for multiple comparisons
☐	☒	A full description of the statistical parameters including central tendency (e.g. means) or other basic estimates (e.g. regression coefficient) AND variation (e.g. standard deviation) or associated estimates of uncertainty (e.g. confidence intervals)
☐	☒	For null hypothesis testing, the test statistic (e.g. F, t, r) with confidence intervals, effect sizes, degrees of freedom and P value noted<br>*Give P values as exact values whenever suitable.*
☒	☐	For Bayesian analysis, information on the choice of priors and Markov chain Monte Carlo settings
☒	☐	For hierarchical and complex designs, identification of the appropriate level for tests and full reporting of outcomes
☒	☐	Estimates of effect sizes (e.g. Cohen's d, Pearson's r), indicating how they were calculated

Our web collection on statistics for biologists contains articles on many of the points above.

Software and code

Policy information about availability of computer code

Data collection	Microsoft Excel Home and Student 2019
Data analysis	GraphPad Prism 9

For manuscripts utilizing custom algorithms or software that are central to the research but not yet described in published literature, software must be made available to editors and reviewers. We strongly encourage code deposition in a community repository (e.g. GitHub). See the Nature Portfolio guidelines for submitting code & software for further information.

Data

Policy information about availability of data

All manuscripts must include a data availability statement. This statement should provide the following information, where applicable:

- Accession codes, unique identifiers, or web links for publicly available datasets
- A description of any restrictions on data availability
- For clinical datasets or third party data, please ensure that the statement adheres to our policy

The source data for Tables and Figures in this study are provided in the Source Data File.

Research involving human participants, their data, or biological material

Policy information about studies with human participants or human data. See also policy information about sex, gender (identity/presentation), and sexual orientation and race, ethnicity and racism.

Reporting on sex and gender	Sex for donors has been collected, as recorded by donation agencies. Gender was not collected but has no influence on data handling or analysis. Sex and gender had no bearing on data analysis or results.
Reporting on race, ethnicity, or other socially relevant groupings	Not collected, therefore not reported. No relevance to data.
Population characteristics	Age was collected from donation agencies.
Recruitment	Consent for research was obtained at the time of consent for organ donation by the centralised donation organisation in Australia, DonateLife. Consent was obtained by independent donation specialists with no relation or influence by the research team.
Ethics oversight	Sydney Local Health District Ethics Review Committee (X18-0523 & 2019/ETH08964)

Note that full information on the approval of the study protocol must also be provided in the manuscript.

Field-specific reporting

Please select the one below that is the best fit for your research. If you are not sure, read the appropriate sections before making your selection.

☒ Life sciences ☐ Behavioural & social sciences ☐ Ecological, evolutionary & environmental sciences

For a reference copy of the document with all sections, see nature.com/documents/nr-reporting-summary-flat.pdf

Life sciences study design

All studies must disclose on these points even when the disclosure is negative.

Sample size	No sample size calculation required. Single intervention group. A total of 10 liver perfusions were performed, with 10 successful splits, which resulted in 20 partial organs. This is the largest sample size in the field. A sample size calculation was not possible as there are no previous studies for comparison. We considered a sample size of 20 organs to be reasonable to produce a proof of concept model.
Data exclusions	No data excluded. Variables that were measured but not contributory were not reported (glucose, pH, INR)
Replication	10 experiments with 20 partial livers. Same conditions replicated in all cases.
Randomization	Not relevant. Single intervention group.
Blinding	Not relevant. Single intervention group.

Reporting for specific materials, systems and methods

We require information from authors about some types of materials, experimental systems and methods used in many studies. Here, indicate whether each material, system or method listed is relevant to your study. If you are not sure if a list item applies to your research, read the appropriate section before selecting a response.

Materials & experimental systems

n/a	Involved in the study	
☒	☐	Antibodies
☒	☐	Eukaryotic cell lines
☒	☐	Palaeontology and archaeology
☒	☐	Animals and other organisms
☒	☐	Clinical data
☒	☐	Dual use research of concern
☒	☐	Plants

Methods

n/a	Involved in the study	
☒	☐	ChIP-seq
☒	☐	Flow cytometry
☒	☐	MRI-based neuroimaging
